# Blueberry Muffin Sign: A Diagnostic Histochemical Clue in Mitochondrial Myopathy

**DOI:** 10.7759/cureus.117236

**Published:** 2026-09-30

**Authors:** Abraham Alhatou, Omar M Alhatou, Anwar I Joudeh, Mohammed Alhatou

**Affiliations:** 1 DeBusk College of Osteopathic Medicine, Lincoln Memorial University, Knoxville, USA; 2 Medicine, Royal College of Surgeons in Ireland - Bahrain, Busaiteen, BHR; 3 Internal Medicine, Qatar University, Doha, QAT; 4 Internal Medicine, Hamad Medical Corporation, Doha, QAT; 5 Neurosciences, Hamad Medical Corporation, Doha, QAT

**Keywords:** blueberry muffin sign, case report, mitochondrial myopathy, muscle biopsy, ophthalmoplegia

## Abstract

Chronic progressive external ophthalmoplegia (CPEO) is a rare manifestation of mitochondrial myopathy, marked by slowly progressive bilateral ptosis and ophthalmoplegia that can mimic other ocular myopathies, often leading to delayed diagnosis. We report a 63-year-old female with a 24-year history of slowly progressive bilateral ptosis, ophthalmoplegia, and proximal muscle weakness. Laboratory investigations, brain and orbital magnetic resonance imaging, and extensive genetic testing were unrevealing, and she did not respond to empirical treatment for presumed myasthenia gravis. Muscle biopsy demonstrated ragged red fibers, increased succinate dehydrogenase (SDH) activity, and cytochrome c oxidase (COX)-deficient fibers appearing intensely blue on combined SDH/COX staining, a pattern the authors term the "Blueberry Muffin Sign." Electron microscopy confirmed paracrystalline "parking lot" inclusions, supporting a diagnosis of CPEO due to mitochondrial myopathy. The "Blueberry Muffin Sign" on combined COX-SDH histochemical staining represents a potentially useful diagnostic clue for mitochondrial myopathy that may facilitate earlier recognition of CPEO, particularly when genetic testing is inconclusive; validation in larger, genetically characterized cohorts is needed to establish its diagnostic specificity and reproducibility.

## Introduction

Mitochondrial myopathies vary in presentation and course from mild exercise intolerance to a fatal multisystemic disease. This clinical heterogeneity is likely due to the diverse mitochondrial and nuclear genes that mitochondrial function depends on. Chronic progressive external ophthalmoplegia (CPEO) is a well-recognized manifestation of mitochondrial disorders. It is characterized by slowly progressive paresis of the extraocular muscles with bilateral ptosis starting in the fourth decade, although it may present at any age. Despite the marked extraocular muscle weakness, diplopia is often absent due to the symmetrical involvement [1].

Diagnosis of CPEO due to mitochondrial myopathy can be challenging, as its clinical presentation overlaps considerably with other neuromuscular and ocular disorders, and genetic testing fails to identify a pathogenic variant in a substantial proportion of affected patients. In such cases, muscle biopsy with histochemical analysis remains central to diagnosis [2]. Combined cytochrome c oxidase (COX) and succinate dehydrogenase (SDH) staining is particularly useful, as it simultaneously demonstrates COX deficiency and compensatory mitochondrial proliferation within the same fibers, producing a distinctive dual-color histochemical pattern [3]. Despite its diagnostic value, this pattern is not always readily recognized by clinicians and pathologists who are less familiar with muscle histopathology. We propose the descriptive term "Blueberry Muffin Sign" to aid recognition of this pattern and illustrate its application in a patient with genetically inconclusive CPEO.

## Case presentation

Case history and examination

A 63-year-old female, born to non-consanguineous parents with no family history of myopathy or neuropathy, presented with a history of slowly progressive bilateral ptosis, ophthalmoplegia, and proximal muscle weakness without fluctuation in symptom severity, with symptoms beginning at approximately 39 years of age. Physical examination showed severe ptosis and global ophthalmoplegia. However, the direct and indirect pupillary reflexes were preserved. Motor exam revealed mild symmetrical weakness of the neck flexors and proximal limb muscles (Medical Research Council grade 4+/5 [4]) with preserved reflexes and sensation.

Differential diagnosis, investigations, and treatment

Laboratory investigations, including creatine kinase, aldolase, thyroid function, lactate, and metabolic profile, were within the normal range. Myositis-specific and connective tissue disease antibody panels, as well as acetylcholine receptor and MuSK antibodies for autoimmune myasthenia gravis, were also negative. Electrodiagnostic studies showed proximal non-irritable myopathic changes without neuromuscular junction dysfunction. Electrocardiography (EKG) was normal. Brain and orbital magnetic resonance imaging were also unremarkable. Extensive genetic testing of peripheral blood and buccal specimens was conducted for a wide range of neuromuscular disorders, including panels for muscular dystrophies, congenital myopathies, congenital myasthenic syndromes, and combined mitochondrial genome analysis, and all were unrevealing. Prior treatment for presumed myasthenia gravis with pyridostigmine and prednisone was ineffective.

Muscle biopsy showed subsarcolemmal mitochondrial accumulation on modified Gomori trichrome staining consistent with ragged red fibers (Figure 1A), increased SDH activity (Figure 1B), and COX-deficient fibers appearing intensely blue on combined SDH/COX staining (Figure 1C). Electron microscopy further confirmed the presence of typical type I "parking lot" paracrystalline inclusions (Figure 2). In the absence of a confirmed genetic abnormality, the diagnosis of mitochondrial myopathy was established on the basis of characteristic histopathological and ultrastructural findings, including ragged red fibers, COX-deficient fibers with compensatory SDH hyperactivity, and paracrystalline "parking lot" inclusions, which are considered diagnostic of mitochondrial myopathy independent of molecular confirmation [2,3,5].

**Figure 1:**
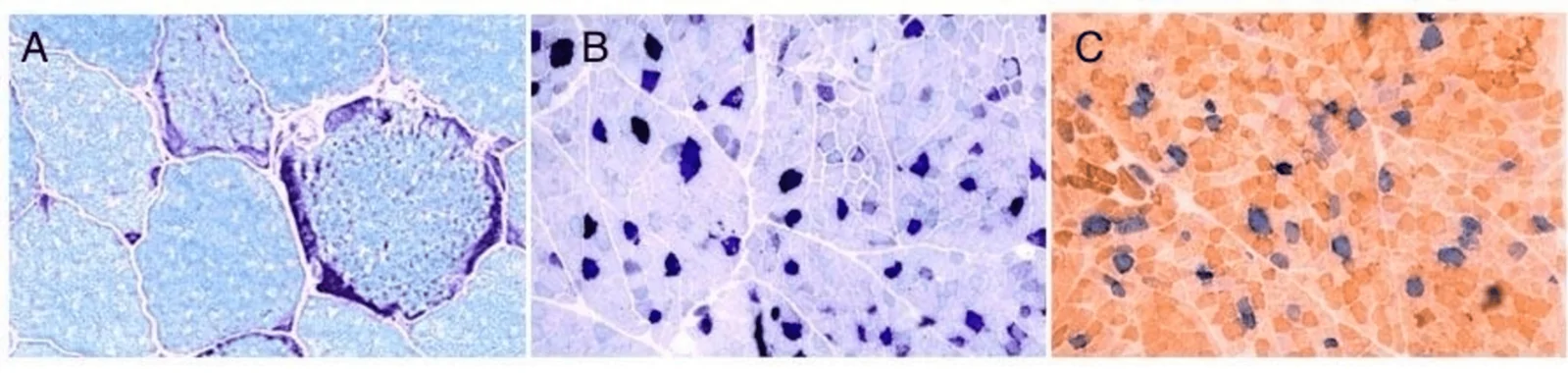
Muscle biopsy histochemistry. Modified Gomori trichrome staining showing ragged red fibers (A), increased mitochondrial activity on succinate dehydrogenase (SDH) staining (B), and intensely blue cytochrome c oxidase (COX)-deficient fibers exhibiting the "Blueberry Muffin Sign" on combined COX-SDH staining (C).

**Figure 2:**
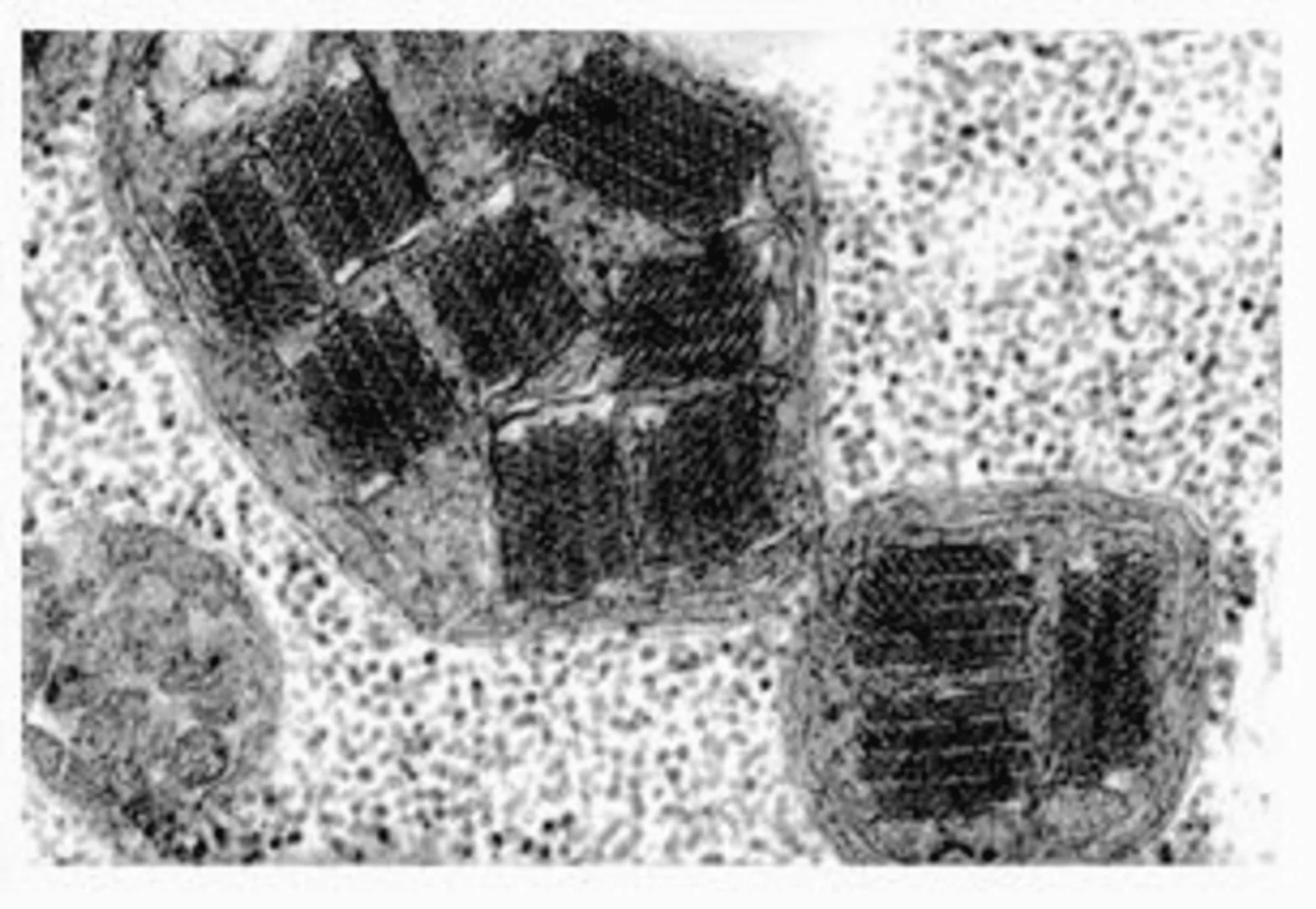
Electron microscopy of muscle biopsy. Electron microscopy showing paracrystalline "parking lot" inclusions within the mitochondria.

Outcome and follow-up

The patient was diagnosed with CPEO due to mitochondrial myopathy. Given the absence of a confirmed genetic etiology, no mitochondrial-directed pharmacologic therapy was initiated, and the patient was managed symptomatically. She was referred for genetic counseling and continued under neurology follow-up.

## Discussion

Delayed diagnosis of CPEO is not uncommon due to similarity in presentation to other ocular conditions such as myasthenia gravis, ocular myositis, and thyroid-related orbitopathy, among others. Therefore, muscle biopsy and histochemical analysis remain essential, particularly when genetic testing is inconclusive. The combined COX-SDH technique enhances detection of respiratory chain defects by simultaneously demonstrating enzyme deficiency and mitochondrial proliferation [3].

The proposed "Blueberry Muffin Sign" provides a simple visual correlation of this dual pathology, distinguishing true COX-negative fibers from partial deficiencies. Similar histochemical patterns have been described in mitochondrial disorders, but this terminology emphasizes its diagnostic utility and recognizability [6]. The specificity of this appearance for mitochondrial myopathy has not been formally established, and comparable COX-deficient, SDH-hyperreactive fibers may potentially be observed in other conditions associated with mitochondrial proliferation or respiratory chain dysfunction; the term is therefore intended primarily as a descriptive and educational aid to pattern recognition rather than as a validated, stand-alone diagnostic test. It should also be emphasized that the diagnosis of mitochondrial myopathy in this case rests on the objective histopathological and ultrastructural findings described above, while the "Blueberry Muffin Sign" refers specifically to the visual appearance of the combined COX-SDH stain. Mechanistically, the COX-deficient component of this pattern reflects dysfunction of respiratory chain complex IV, which is partly mitochondrial DNA (mtDNA)-encoded, whereas the preserved SDH staining reflects intact respiratory chain complex II, which is nuclear DNA-encoded; a mosaic distribution of COX-deficient fibers against this preserved SDH background is therefore particularly suggestive of an underlying mtDNA defect [7,8]. Recognition of this sign may nonetheless facilitate earlier diagnosis of mitochondrial myopathy, particularly in CPEO phenotypes and seronegative "myasthenia-like" presentations. Incorporating such visual descriptors into routine neuropathology practice may improve diagnostic accuracy and clinical correlation.

The diagnostic delay observed in our patient, spanning 24 years, mirrors recent evidence that CPEO is frequently misdiagnosed. In a cohort of patients ultimately confirmed to have CPEO, Karimi et al. reported that a substantial proportion had initially been labeled with alternative diagnoses, most commonly ocular myasthenia gravis, aponeurotic blepharoptosis, or thyroid eye disease, with a considerable time lag before the correct diagnosis was established [9]. This pattern parallels our patient's initial presumptive diagnosis of, and empiric treatment for, myasthenia gravis, which proved ineffective. Comparable diagnostic pitfalls have been described when CPEO coexists with, or is overshadowed by, other neuro-ophthalmologic findings, reinforcing the need for a high index of suspicion when chronic ptosis and ophthalmoplegia fail to respond to standard immunomodulatory therapy [10].

The negative genetic testing in this patient, despite an extensive panel covering muscular dystrophies, congenital myopathies, congenital myasthenic syndromes, and combined mitochondrial genome analysis, highlights an important diagnostic limitation. Current clinical panels may not capture all pathogenic variants, particularly given the possibility of low-level heteroplasmy, tissue-specific mosaicism, or variants in genes not yet included in standard panels [3]. Such low-level or tissue-specific heteroplasmy is itself a recognized feature of mtDNA-related disease and can render peripheral blood or buccal genetic testing falsely reassuring despite genuine underlying mtDNA pathology [7,8]. In such cases, histochemical and ultrastructural findings remain the diagnostic gold standard, providing morphological evidence of mitochondrial dysfunction independent of a confirmed genetic etiology. This case underscores that a negative genetic result should not preclude further morphological evaluation when clinical suspicion for mitochondrial disease remains high.

The Mitochondrial Medicine Society's consensus guidelines note that tissue biopsy is indicated specifically when a diagnosis cannot be confirmed by DNA testing and that histochemical and biochemical assessment of muscle can demonstrate oxidative phosphorylation defects even when nuclear and mitochondrial genome sequencing is negative [5]. Contemporary reviews of mitochondrial CPEO, however, increasingly frame genetic sequencing itself as the diagnostic gold standard, reserving muscle biopsy for cases in which molecular testing is unrevealing or inconclusive [11]. Our case illustrates this complementary relationship: despite a combined mitochondrial genome and multigene nuclear panel, the diagnosis was established only through muscle histochemistry and ultrastructural analysis. This experience reinforces the recommendation that a normal or inconclusive genetic panel should prompt tissue-based evaluation rather than exclusion of mitochondrial disease, particularly in patients with a clinically compatible phenotype.

This report is limited by its description of a single patient, which precludes generalization of the "Blueberry Muffin Sign" as a universally reliable diagnostic marker. Further studies correlating this histochemical pattern across a larger cohort of genetically confirmed and unconfirmed mitochondrial myopathy cases would help validate its diagnostic utility.

## Conclusions

Delayed diagnosis of CPEO is not uncommon, particularly when the clinical presentation is restricted or atypical. Inconclusive genetic testing is similarly common in this setting, as low-level mutant heteroplasmy characteristic of mtDNA-related disease can escape detection on standard panels. This case underscores the diagnostic value of muscle biopsy and histochemical analysis. The presence of COX-deficient fibers, reflecting complex IV (mtDNA-encoded) dysfunction, together with preserved SDH staining, reflecting intact complex II (nuclear DNA-encoded) activity, is strongly suggestive of an underlying mtDNA defect. The combined COX-SDH technique enhances detection of such abnormalities, rendering them more visually distinct and less likely to be overlooked.

We propose the term "blueberry muffin sign" to describe this composite histochemical pattern. While similar histochemical patterns have been described in other mitochondrial disorders, this terminology is intended to improve recognizability and diagnostic utility in routine practice. Incorporating such visual descriptors into routine neuropathology reporting may enhance diagnostic accuracy and clinicopathological correlation.
